# Baseline status of policy and legislation actions to address non communicable diseases crisis in the Pacific

**DOI:** 10.1186/s12889-020-08795-2

**Published:** 2020-05-12

**Authors:** Si Thu Win Tin, Ilisapeci Kubuabola, Amerita Ravuvu, Wendy Snowdon, A. Mark Durand, Paula Vivili, Erin Passmore

**Affiliations:** 1 Public Health Division, Pacific Community (SPC), Suva, Fiji; 2 The Boden Institute, the Sydney Medical School, The University of Sydney, Sydney, Australia; 3 Pacific Research Centre for the Prevention of Obesity and Non-Communicable Diseases (C-POND), College of Medicine, Nursing & Health Sciences, Fiji National University, Suva, Fiji; 4 Division of Pacific Technical Support, World Health Organisation (WHO), Suva, Fiji; 5 Pacific Islands Health Officials Association (PIHOA), Honolulu, HI USA; 6 Public Health, Division, Pacific Community (SPC), Noumea, New Caledonia

**Keywords:** Non-communicable diseases, Pacific, Policy, Legislation, Dashboard, Accountability

## Abstract

**Background:**

Non-Communicable Diseases (NCD) are the leading cause of death in the Pacific Island Countries and Territories (PICTs) accounting for approximately 70% of mortalities. Pacific leaders committed to take action on the Pacific NCD Roadmap, which specifies NCD policy and legislation. To monitor progress against the NCD Roadmap, the Pacific Monitoring Alliance for NCD Action (MANA) was formed and the MANA dashboard was developed. This paper reports on the first status assessment for all 21 PICTs.

**Methods:**

The MANA Dashboard comprises 31 indicators across the domains of leadership and governance, preventive policies, health system response and monitoring processes, and uses a ‘traffic light’ rating scheme to track progress. The dashboard indicators draw on WHO’s best-buy interventions and track highly cost-effective interventions for addressing NCDs. The MANA coordination team in collaboration with national NCD focal points completed Dashboards for all 21 PICTs between 2017 and 2018 in an agreed process. The data were analysed and presented within each area of the MANA dashboard.

**Results:**

This assessment found that PICTs are at varying stages of developing and implementing NCD policy and legislation. Some policy and legislation are in place in most PICTs e.g. smoke free environment (18 PICTs), alcohol licensing (19 PICTs), physical education in schools (14 PICTs), reduction of population salt consumption (14 PICTs) etc. However, no PICTs has policy or legislation on tobacco industry interference, controlling marketing of foods and drinks to children, and reducing trans-fats in the food supply, and only 7 PICTs have policies restricting alcohol advertising. Eighteen PICTs implement tobacco taxation measures, however only five were defined as having strong measures in place. Nineteen PICTs have alcohol taxation mechanisms and 13 PICTs have fiscal policies on foods to promote healthier diets.

**Conclusion:**

This baseline assessment fills a knowledge gap on current strengths and areas where more action is needed to scale up NCD action in a sustained ‘whole of government and whole of society approach’ in PICTs. The findings of this assessment can be used to identify priority actions, and as a mutual accountability mechanism to track progress on implementation of NCD policy and legislation at both national and Pacific level.

## Background

The global burden of Non Communicable Diseases (NCD) is increasing, and is a major threat to health and sustainable development [1, 2]. Premature death, disability and reduced productivity from NCD pose a heavy burden on governments, communities, families and individuals [1]. The Pacific Island Countries and Territories (PICTs) have some of the highest rates of NCD and associated risk factors in the world. NCD are the leading cause of death in most PICTs [3], accounting for approximately 70% of mortalities [4] and creating a ‘human, social and economic crisis’ that challenge to achieve Healthy Island Vision [5] and Sustainable Development Goals [6].

Recognising this, at the Joint Forum Economic and Health Ministers Meeting in 2014, Pacific ministers endorsed the Pacific NCD Roadmap [4] and committed to take action at both national and regional levels. The Roadmap specifies policy and legislation measures aimed at preventing NCD and includes a menu of over 30 other multi-sectoral interventions suited to PICTs. To monitor progress against the Roadmap, the Pacific Monitoring Alliance for NCD Action (MANA) was formed, and a mutual accountability mechanism using the MANA dashboard was developed [7]. The MANA Dashboard incorporates and expands on the set of indicators used for the World Health Organization NCD Progress Monitor, and the Dashboard is used to assess the status of NCD policy and legislation in PICTs. The Pacific Heads of Health and Health Ministers requested status and annual update reports on progress against the MANA dashboard at all future meetings to promote accountability and ultimately promote stronger NCD action across the Pacific, with the first completed one to be presented in 2019. This paper reports on the first status assessment for all 21 PICTs.

## Methods

This status assessment using MANA dashboards was conducted between 2017 and 2018 for 21 PICTs. These include American Samoa, Commonwealth of the Northern Mariana Islands, Cook Islands, Federated States of Micronesia, Fiji, French Polynesia, Guam, Kiribati, Nauru, Niue, New Caledonia, Palau, Papua New Guinea, Republic of the Marshall Islands, Tokelau, Tonga, Samoa, Solomon Islands, Tuvalu, Vanuatu, and Wallis and Futuna.

The MANA dashboard comprises 31 NCD indicators covering four categories [7]. These include the domains on leadership and governance (e.g. existence of a multi-sectoral NCD taskforce, national strategies addressing NCD and risk factors, and national NCD targets); preventive policies (e.g. for tobacco, alcohol, food environments and physical activity); health system response programmes (e.g. access to NCD treatment and drugs, tobacco cessation programmes, and maternal and infant nutrition initiatives); and routine monitoring processes (e.g. adult and adolescent risk factor surveys, child growth monitoring and NCD-related mortality). The dashboard uses a ‘traffic light’ rating scheme to track progress: red for no policy/action present; amber for policy/action under development; and green for policy/action in place [7]. When a policy/action is in place (green), the strength of the actions is assessed using a star system (zero to three stars) (Table 1).

**Table 1:**
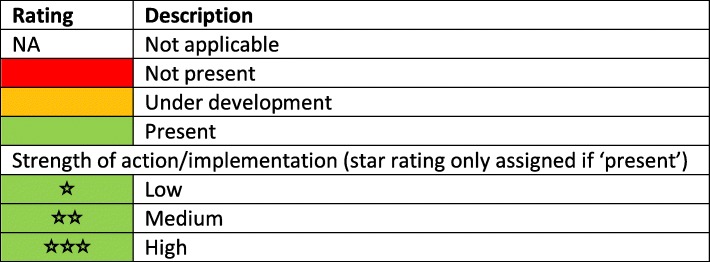
Key for indicator ratings for the Pacific MANA Dashboard

Indicator definitions and criteria (See Additional file 1: Pacific MANA Dashboard Data Dictionary) for assessing the strength of action for each indicator were developed, refined and piloted by the MANA coordination team, and endorsed by the Pacific Heads of Health and Health Ministers in 2017 [8]. Most indicators were based on existing global indicators from the World Health Organization’s NCD Progress Monitor [3] which track highly cost-effective interventions for addressing NCDs, with scoring criteria adapted to reflect the ‘traffic light’ scoring system. Some indicators were newly-developed by the MANA coordination team to complement these existing indicators. The MANA coordination team includes NCD policy experts from the Pacific Community, World Health Organization, Pacific Islands Health Officers’ Association and the Pacific Centre for Prevention of Obesity at the Fiji National University.

Between 2017 and 2018, the Pacific MANA coordination team members liaised with NCD focal points in all 21 PICTs to complete the dashboards [9]. Members of the coordination team first pre-filled the dashboard using publicly available information. The draft dashboards were reviewed, amended and verified with supporting documentation by national NCD focal points of all 21 PICTs. The dashboards were cross-checked by the MANA coordination team to ensure consistent interpretation of indicators across countries, and endorsed by the Minister of Health or other appropriate signatories from each PICT. The data were analysed and compiled in 2018 using Microsoft Excel 2016, and presented within each category of MANA dashboard.

## Results

The following summarises the findings for the 31 indicators across four different domains of the MANA dashboards for all 21 PICTs.

### Leadership and governance

Fourteen of the 21 PICTs have a current national multi-sectoral NCD strategy. Of these, nine PICTs were rated as having ‘strong’ (i.e. three stars green rating) strategy in place. Fifteen PICTs have established NCD target indicators with nine PICTs rated as ‘strong’. However, only five PICTs (24%) (Guam, Palau, Samoa, Tonga and Tuvalu) have an active multi-sectoral NCD taskforce that oversee the implementation of their national multi-sectoral NCD strategy (Table 2).

**Table 2:**
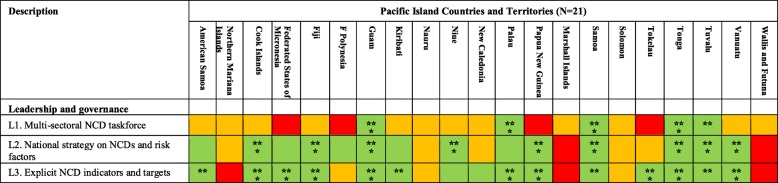
PICT ratings for Pacific MANA Dashboard leadership and governance indicators

### Preventive policies and legislations

#### Taxation measures

Most PICTs have implemented one or more taxation measures on unhealthy products. Eighteen PICTs implement tobacco taxation measures, however only five (24%) (French Polynesia, New Caledonia, Palau, Tonga, and Wallis and Futuna) were rated as having strong measures in place i.e. ‘three stars green rating’. Nineteen PICTs have alcohol taxation mechanisms, however only four PICTs (19%) (Fiji, Nauru, New Caledonia and Tuvalu) have ‘strong’ measures in place. Thirteen PICTs have fiscal policies in place to promote healthier eating, such as taxation on sugar sweetened beverages (SSB) and unhealthy foods, and tax exemptions for fruit and vegetable imports. However only four PICTs (19%) (Kiribati, Nauru, Samoa and Tonga) were rated as having ‘strong’ measures in place (Table 3).

**Table 3:**
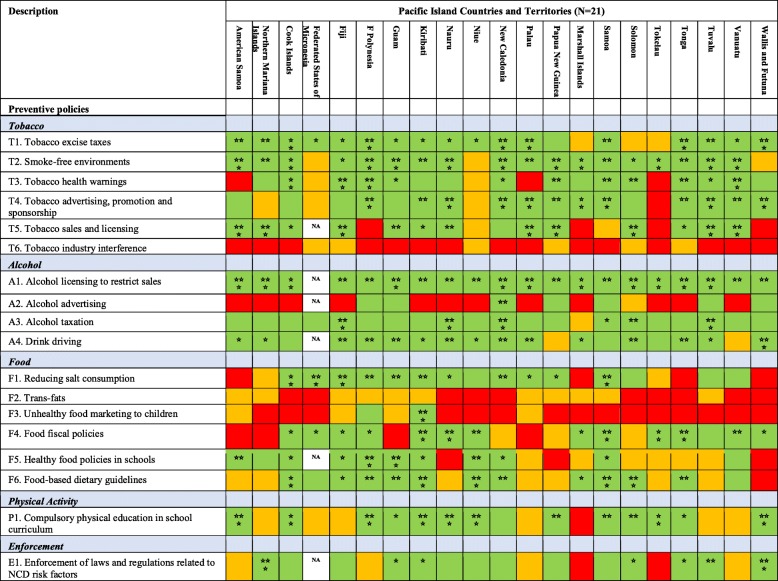
PICT ratings for Pacific MANA Dashboard preventive policy indicators

#### Tobacco and alcohol

Most PICTs have legislation to create smoke-free public places (18 PICTs), health warnings on tobacco packaging (16 PICTs), restrictions on tobacco sales and licensing (14 PICTs), and restrictions on tobacco advertising (17 PICTs). Most countries have national licensing regulations in place to restrict the sale of alcohol (e.g. restriction the hours and locations of sales), and most have legislation to control drink driving. However, the strength of actions for tobacco and alcohol control indicators varied greatly among countries (Table 3).

#### Diet and physical activity

Fourteen PICTs have programmes or policies to reduce population salt consumption, 13 have national food-based dietary guidelines, two have policies to restrict marketing of foods to children, and 11 have policies/guidelines on food in schools. Fourteen countries have included physical activity as a compulsory component of the school curriculum. While some PICTs have strong actions that address diet and physical activity policy, many were rated as being of low strength (i.e. no or one star green rating) (Table 3).

#### Enforcement

Fourteen PICTs have a government-level system in place to support enforcement of tobacco, alcohol, food and/or betel nut legislation. However, the strength of enforcement systems were weak with only two PICTs (10%) (Commonwealth of the Northern Mariana Islands, and Wallis and Futuna) rated as having strong systems in place (Table 3).

### Health system response programmes

Most PICTs have national guidelines in place for the diagnosis and management of at least one of the four main NCD. Fifteen of the 21 PICTs have all essential NCD medicines included in the national list of essential medicines. Smoking cessation support is available in 15 PICTs. Seven PICTs have restrictions on the marketing of breast milk substitutes, six PICTs have a public hospital which has previously been certified as a baby friendly hospital, and eight PICTs have legislation in place providing at least 12 weeks paid maternity leave. The strengths of actions for health system response programmes varied across PICTs (Table 4).

**Table 4:**
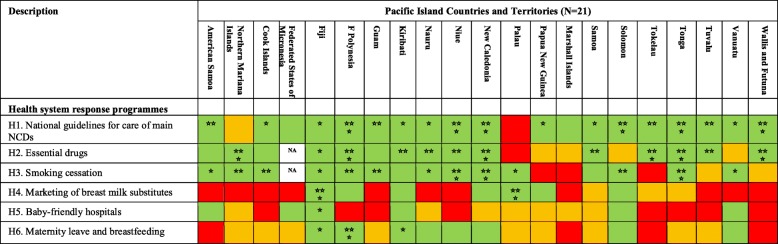
PICT ratings for Pacific MANA Dashboard health system response indicators

### Monitoring

Sixteen PICTs have established mechanisms to monitor NCD risk factor prevalence in adults, typically via population-based surveys such as the WHO STEPwise approach to Surveillance (STEPS), and have collected data within the last five years. Fourteen PICTs have collected adolescent NCD risk factor prevalence data in the last five years, typically via school-based surveys such as the Global School-based Student Health Survey and the Global Youth Tobacco Survey. Eleven PICTs have collected and reported data on child growth. Most have established systems for routinely reporting cause-specific mortality with 11 PICTs (American Samoa, Commonwealth of the Mariana Islands, Cook Islands, French Polynesia, Guam, Kiribati, Nauru, New Caledonia, Palau, Samoa and Tuvalu) rated as ‘strong’ (Table 5).

**Table 5:**
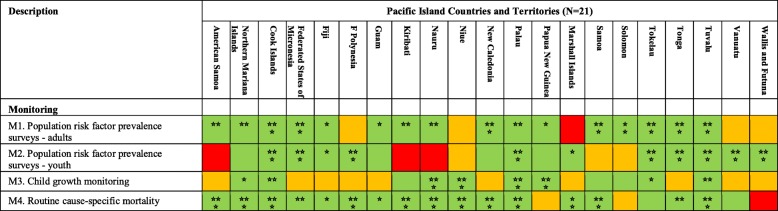
PICT ratings for Pacific MANA Dashboard monitoring indicators

## Discussion

This study found that PICTs are at varying stages of developing and implementing NCD policy and legislation. While some NCD policy and legislation are in place in most PICTs (e.g. smoke free environment, alcohol licensing etc.), there are several policy and legislation gaps that need urgent attention to scale up NCD action across the Pacific. These include tobacco industry interference, alcohol advertising, limiting of trans-fats, marketing of foods and drinks to children, marketing of breastmilk substitutes and the certification of baby-friendly hospitals.

The critical role of policy and legislation to address NCD has been widely acknowledged by global and regional leaders [2, 4]. Taxes and subsidies can incentivise healthy lifestyle behaviours, and can generate revenue that can be invested in prevention and control of NCD efforts at the national and community level [10–12]. Evidence has shown that high level political leadership, good governance, strong policies and systems can promote health and prevent diseases [12].

Despite commitments made at regional level [4], there is still a need to strengthen multi-sectoral leadership and governance at national level. For example, this assessment found that only a few PICTs have functioning national multi-sectoral taskforce to lead implementation of their multi-sectoral national NCD plan. This may be due to competing priorities or lack of commitment from different sectors at the national level. Since NCD are driven by multiple factors, both within and outside the health sector, an active multi-sectoral NCD strategy with clearly defined target indicators monitored through national taskforces are essential to effectively address NCD. Health-in-all-policies, through a whole-of-government and whole-of-society approach is needed to combat NCDs [13].

Most PICTs have taxation measures on unhealthy products in place; however, there is a need to further increase taxes in line with global recommendations [14]. This requires commitment and collaborative efforts from health, trade and finance ministries. Pacific leaders have already committed to taking action on tobacco as part of Tobacco Free Pacific 2025 [15] and WHO Framework Convention for Tobacco Control (FCTC) commitments [16]. This assessment result has shown that preventing tobacco industry interference is a key policy gap in PICTs, requiring urgent national action. Industry interference can thwart efforts to strengthen policy and legislation [17]. Other challenges that need urgent national attention include limiting trans-fats in the food supply, regulating alcohol advertising, and more importantly, enforcement of policies and legislations. Banning trans-fats is recommended by WHO as a cost-effective intervention [18], given the evidence that eliminating trans-fats from the food supply is expected to impact directly on cardiovascular disease mortality [19, 20].

Restricting marketing of foods and drinks to children is also a key gap in PICTs. The report of the WHO Commission on Ending Childhood Obesity [12] recommends implementing the set of recommendations on the marketing of foods and non-alcoholic beverages to children [21] to reduce the power of the marketing of foods and the exposure of children and adolescents to it. In addition, there are areas where very few PICTs have established policy to address childhood obesity, for example, restricting marketing of breast milk substitutes, baby friendly hospitals accreditation, and provision of breastfeeding facilities. Multi-sectoral collaboration to implement childhood obesity policies involving health, education, law enforcement, trade and other ministries have not yet been achieved in most PICTs.

While ensuring key NCD medications are included in the Essential drug lists within countries is a critical first step to managing NCDs, it is critical that such medicines are continuously available without stock-outs, and this remains a challenge in some PICTs.

Despite most PICTs having completed at least one adult and one adolescent NCD risk factor survey, further action is still required to ensure that surveys are scheduled frequently enough to monitor population trends and guide interventions. There is also a need to strengthen mechanisms for reporting of child growth monitoring to better monitor trends in child over- and underweight in PICTs.

Although the dashboards identify important information, the results are for the dashboards endorsed in 2017–2018 and will be updated by the end of 2019 to reflect changes in policy and legislation since the completion and endorsement of the dashboards. As this was the first round of data collection and validation using MANA dashboards, it was more time-consuming than is expected in subsequent rounds, when the process will only be to update existing dashboards annually. The dashboard indicators will also be subject to ongoing review, and may be refined to reflect emerging health priorities or new data sources.

## Conclusion

This baseline assessment fills a knowledge gap by providing an overview of the status of policy and legislation in PICTs and will serve as a baseline for tracking policy change over time. It also provides useful information on current strengths and areas where more action is needed to effectively address social, economic, environmental and commercial determinants of NCD in a sustained ‘whole of government and whole of society approach’. The findings of this assessment can be used to identify national priority actions and to track progress on NCD policy and legislation. At the regional level, dashboards can be used as a mutual accountability mechanism to monitor and update PICTs progress on NCD action annually through Pacific Heads of Health, Health Ministers and Economic Ministers Meetings. This accountability mechanism will strongly contribute to scaling up of NCD action towards achieving Sustainable Development Goals and the Healthy Island Vision.

## Supplementary information

**Additional file 1.** Pacific Monitoring Alliance for Non-Communicable Diseases Action (MANA) Dashboard Data Dictionary.

## Data Availability

All data generated or analysed during this assessment are included in this article.

## Abbreviations

C-POND: Pacific Research Centre for Prevention of Obesity and Non Communicable Diseases
MANA: Pacific Monitoring Alliance for Non Communicable Diseases Action
NCD: Non Communicable Diseases
PICTs: Pacific Islands Countries and Territories
PIHOA: Pacific Islands Health Officials Association
SPC: Pacific Community
STEPS: World Health Organization STEPwise approach to Surveillance
WHO: World Health Organization
