# Supplementary material for: Baseline status of policy and legislation actions to address non communicable diseases crisis in the Pacific
Source: BMC Public Health. 2020 May 12;20:660. doi: 10.1186/s12889-020-08795-2 (PMC7216373; doi:10.1186/s12889-020-08795-2)
Supplement: Supplementary file 1 — Additional file 1. Pacific Monitoring Alliance for Non-Communicable Diseases Action (MANA) Dashboard Data Dictionary. [file 12889_2020_8795_MOESM1_ESM.pdf]

Additional File 1: Pacific Monitoring Alliance for Non-Communicable Diseases Action (MANA) Dashboard Data Dictionary

Key

N/A	Not applicable
(red)	Not present
(amber)	Under development
(green)	Present
Strength of action/implementation (star rating only assigned if 'Present')	
☆	Low
☆ ☆	Medium
☆ ☆ ☆	High

Leadership and governance

L1. Multi-sectoral NCD taskforce

A multi-sectoral taskforce is operating, reports regularly, is inclusive of all relevant stakeholders, and is catalysing and monitoring actions on NCDs
WHO Equivalent Indicator: No equivalent

(red)	A multi-sectoral NCD taskforce covering the four main NCD risk factors (tobacco, alcohol, nutrition, physical activity) has not been established, or is inactive (less than two meetings in the last 12 months)
(amber)	There is evidence that a multi-sectoral NCD taskforce is being established, or a taskforce exists and has had at least two meetings in the last 12 months but no public reports are available
(green)	A multi-sectoral NCD taskforce has had at least two meetings in the last 12 months, and an annual report (or equivalent) is available
☆	As for (green), and one of the items listed below
☆ ☆	As for (green), and three of the items listed below
☆ ☆ ☆	As for (green), and four or more of the items listed below
	• The taskforce is led by a government minister or prime minister<br>• The NCD taskforce demonstrates decision-making, monitors implementation and publicly documents its actions.<br>• The taskforce includes senior representation from government sectors, such as: attorney general, and ministries of agriculture, communications, customs and excise, education, finance and economic planning, health, labour and industry, sport, national statistics, trade, police, urban planning and national statistics office (at least five)<br>• The taskforce includes civil society and non-governmental organisations<br>• Platform has established mechanisms for engagement with the private sector (with conflicts of interest managed), EXCLUDING the tobacco industryPrivate sector engagement can be through the taskforce or at national level

L2. National strategy addressing NCDs and risk factors

A comprehensive, multi-sectoral national strategy addressing NCDs and risk factors is operational
WHO Equivalent Indicator #4

	There is no current national multi-sectoral strategy for tackling NCDs
	There is evidence that a national multi-sectoral strategy is under development OR one exists but is not operational
	A multi-sectoral NCD strategy has been developed (either stand-alone or part of a wider national health plan) to cover at least two individual diseases (cardiovascular disease, diabetes, cancer, respiratory disease) and two risk factors (tobacco, alcohol, nutrition, physical activity), AND is operational
☆	A multi-sectoral NCD strategy has been developed, is operational, and covers at least four individual diseases and four risk factors
☆ ☆	As for ☆, and one of the items listed below
☆ ☆ ☆	As for ☆ and demonstrates engagement of non-health agencies in development of the strategy, has a monitoring and surveillance plan, and one other item from the list below
	• Includes a comprehensive set of policies and actions translated from agreed global, regional and national frameworks<br>• Evident responsibilities, timelines and accountability mechanisms<br>• Evident budget allocations (in plans or government budgets)<br>• Evident monitoring and surveillance plan

L3. Explicit NCD indicators and targets

Explicit time-bound targets and indicators have been established for national NCD strategy
WHO Equivalent Indicator #1

	There are no current national targets for tackling NCDs
	National quantitative targets and indicators are under development
	Time-bound indicators and targets cover NCD risk factors, NCD prevalence and NCD actions (e.g. policy change)
☆	As for , and covers two, three or four of the WHO global targets (listed below)
☆ ☆	As for , and covers five or more of the WHO global targets
☆ ☆ ☆	As for , and covers five or more of the WHO global targets, and there is a documented plan for reporting (e.g. national NCD strategy has a surveillance and monitoring plan)
	WHO nine global targets:<br>• Risk factors:<br>➤ reduce harmful use of alcohol<br>➤ reduce physical inactivity<br>➤ reduce salt /sodium intake<br>➤ reduce tobacco use<br>➤ reduce raised blood pressure<br>➤ no increase in diabetes/obesity<br>• Health system response<br>➤ 50% coverage for drug therapy and counselling<br>➤ 80% coverage essential NCD drugs and technologies<br>• Mortality<br>➤ reduce premature mortality from NCDs

Preventive policies

Tobacco

T1. Tobacco excise taxes

Legislation is in place to reduce affordability of tobacco products by increasing tobacco excise taxes
WHO Equivalent Indicator #5a

(red)	No excise tax is collected on cigarettes
(amber)	Tobacco excise tax legislation is being developed, or cigarette excise tax ≤ 20% of retail price
(green)	21–30% of retail price of cigarettes is excise tax
☆	31–50% of retail price of cigarettes is excise tax
☆ ☆	51–69% of retail price of cigarettes is excise tax
☆ ☆ ☆	≥70% of retail price of cigarettes is excise tax
	Data for this indicator are obtained from the WHO Report on the Global Tobacco Epidemic, which is published every two years. http://www.who.int/tobacco/global_report/2015/en/<br><br>For PICTs not covered in the WHO Report on the Global Tobacco Epidemic, this indicator was calculated by the MANA Coordination Team using the same method as used in the report, i.e.:<br><br>Specific excise amount ($) / cost per pack ($)<br><br>Denominator for specific excise / number of cigarettes per pack<br><br>For example, if the most popular brand retails for $28.50 per pack of 20 cigarettes and the excise rate is $494 per 1,000 cigarettes, excise tax as a proportion of retail price = (494/28.50)/(1,000/30) = 52%<br><br>Cost per pack: This is the tax-inclusive retail sales price in local currency per pack of 20 sticks, of the most popular brand of cigarettes, the brand as determined by the country NCD focal point. The retail price is calculated as the average of the retail prices from at least three different locations (locations include a mix of shop sizes, e.g. supermarket, petrol station, small family-owned shop).

T2. Smoke-free environments

Legislation is in place to create public places that are completely smoke-free environments
WHO Equivalent Indicator #5b

(red)	No legislation for smoke-free environments
(amber)	Legislation for smoke-free environments is being developed or currently covers only one area listed below
(green)	Smoke-free environment legislation covers two areas listed
☆	Smoke-free environment legislation covers three areas listed
☆ ☆	Smoke-free environment legislation covers four to seven areas listed
☆ ☆ ☆	Smoke-free environment legislation covers eight areas listed
	Completely smoke-free places include:<br><br>• health-care facilities<br>• educational facilities other than universities<br>• universities<br>• government facilities<br>• indoor offices and workplaces not considered in any other category<br>• restaurants or facilities that serve mostly food<br>• cafes, pubs and bars or facilities that serve mostly beverages<br>• public transport

T3. Tobacco health warnings

Health warnings are in place to warn of the dangers of tobacco and tobacco smoke
WHO Equivalent Indicator #5c

(red)	No legislation requiring health warnings and/or no health warnings on tobacco products
(amber)	Tobacco control legislation and/or health warnings are being developed
(green)	Average proportion of principal display (front and rear combined) mandated to be covered by health warnings is less than or equal to 50%, and no pictorials and no principal language(s)
☆	Average principal display less than or equal to 50%, with pictorials **or** principal language(s)
☆ ☆	Average principal display less than or equal to 50%, with pictorials **and** principal language(s)
☆ ☆ ☆	Average principal display 51% or greater, with pictorials and principal language(s)

T4. Tobacco advertising, promotion and sponsorship

Measures are in place to ban all forms of tobacco advertising, promotion and sponsorship
WHO Equivalent Indicator #5d

(red)	No legislation prohibiting tobacco advertising, promotion and sponsorship
(amber)	Legislation prohibiting tobacco advertising promotion and sponsorship is being developed
(green)	Legislation exists governing standards of tobacco advertising, promotion and sponsorship in at least two areas of **direct** advertising
☆	Legislation completely bans advertising on national television and radio, local magazines and newspapers, billboards/outdoor advertising, and at point of sale
☆ ☆	As for ☆, and at least two other areas of direct or indirect advertising are banned
☆ ☆ ☆	Legislation completely bans ALL forms of direct and indirect advertising listed
	Direct advertising:<br>• national television and radio<br>• local magazines and newspapers<br>• billboards, outdoor advertising<br>• point of sale<br>• retailers and sellers of tobacco must store all tobacco products out of sight<br><br>Indirect advertising:<br>• free distribution of tobacco products in the mail or through other means<br>• promotional discounts<br>• non-tobacco goods and services identified with tobacco brand names (brand extension)<br>• brand names of non-tobacco products used for tobacco products (brand-sharing)<br>• sponsored events, including corporate social responsibility programmes<br>• appearance of tobacco brands or products in television and/or films (product placement)

T5. Tobacco sales and licencing

Measures are in place restricting tobacco sales and licencing
WHO Equivalent Indicator: No equivalent

	No measures are in place restricting tobacco sales and licencing
	Legislation for tobacco sales and licensing are under development
	The sale of single stick cigarettes or loose tobacco is banned
☆	As for , and legislation covers one or two areas listed
☆ ☆	As for , and legislation covers three areas listed
☆ ☆ ☆	As for , and legislation covers four areas listed
	• A licence is required for all manufacturers (where applicable) and importers of tobacco products<br>• A licence is required for all distributors of tobacco products<br>• A license is required for all wholesaler and retailers of tobacco products<br>• Tobacco sales to minors (as defined by the government) are banned

T6. Tobacco industry interference

Government-level policies or laws are in place to prevent tobacco industry interference
WHO Equivalent Indicator: No equivalent

	No government-level tobacco industry interference preventative policies or laws are in place
	Government-level tobacco industry interference preventative policies or laws are planned
	Government-level tobacco industry interference preventative policies (e.g. code of conduct) or laws cover one of the areas listed
☆	Government-level policy or law covers two of the areas listed
☆ ☆	Government-level policy or law covers three of the areas listed
☆ ☆ ☆	Government-level policy or law covers four of the areas listed
	• Requiring transparency by public officials and civil servants when interaction with tobacco industry is necessary<br><br>• Requiring candidates for public office, public officials and civil servants to disclose any potential conflicts of interest related to tobacco control<br><br>• Disallowing government, public officials and civil servants from accepting any type of gift or contribution (from the tobacco industry (Exceptions: compensations due to legal settlements or mandated by law or legally binding and enforcement agreements)<br><br>• Prohibiting public disclosure of activities or expenditure described as 'socially responsible' by the tobacco industry

Alcohol

Alcohol licencing to restrict sales

A1. Licencing regulations are in place to restrict sales of alcohol

WHO Equivalent Indicator #6a

(red)	No licencing regulations are in place to limit the sale of alcohol
(amber)	Alcohol licencing regulations are under development to limit the sale of alcohol
(green)	Alcohol licencing regulations exist to limit the sale of alcohol and cover one of the areas listed
☆	Alcohol licencing regulations cover two of the areas listed
☆ ☆	Alcohol licencing regulations cover three of the areas listed
☆ ☆ ☆	Alcohol licencing regulations cover four of the areas listed, and the minimum age to purchase or be served alcohol is 21
	• A licensing system or monopoly exists on retail sales of beer, wine and spirits<br>• Restrictions exist for on- and off-premise sales of beer, wine and spirits regarding hours and locations of sales **and** restrictions exist for off-premise sales of beer, wine and spirits regarding days of sales<br>• Minimum age to purchase or be served alcohol (beer wine spirits) is 18+ years (The alcohol sales licence stipulates who alcohol can be sold to and/or who is allowed on the premises)<br>• All alcohol producers, importers and wholesalers must hold a licence

A2. Alcohol advertising

Regulations for alcohol advertising are in place, with a system to detect infringements
WHO Equivalent Indicator #6b

(red)	No alcohol advertising regulations are in place
(amber)	Alcohol advertising regulations are under development
(green)	Some alcohol advertising regulations exist
☆	Restrictions exist on alcohol advertising for beer, wine and spirits through all national broadcasting (TV, radio, print and cinemas)
☆ ☆	As for ☆, and restrictions exist for alcohol advertising on outdoors billboards and/or sponsorship of cultural, sports and other events
☆ ☆ ☆	As for ☆ ☆, and a detection system exists for infringement of marketing restrictions

A3. Alcohol taxation

An inflation-adjusted alcohol excise taxation system on beer wine and spirits is in place
WHO Equivalent Indicator #6c

(red)	No alcohol excise tax is collected
(amber)	Alcohol excise taxation is being developed, based on beverage type or ethanol content
(green)	Alcohol excise taxation system is in place and is based on beverage type or ethanol content
☆	Excise tax is based on ethanol content and is applied across all beverage types, OR if bands are applied, excise tax is based on the ethanol content at the top of each band<br>AND<br>Excise tax is reviewed or adjusted for inflation annually for **at least one** beverage type
☆ ☆	Excise tax is based on ethanol content and is applied across all beverage types OR if bands are applied, excise tax is based on the ethanol content at the top of each band<br>AND<br>Excise tax is reviewed annually or adjusted for inflation annually for **ALL** beverage types
☆ ☆ ☆	As for ☆ ☆<br><br>AND<br><br>Excise tax is stated by the government as an important public health tool to reduce alcohol consumption/ harm

A4. Drink driving

Regulations are in place to control drink driving
WHO Equivalent Indicator: No equivalent

(red)	No drink drive regulations are in place
(amber)	Drink drive<br><br>regulations are being developed
(green)	Drink drive regulations are in place and set a maximum blood/breath alcohol content
☆	Regulation covers one of the areas listed
☆ ☆	Regulation covers two of the areas listed
☆ ☆ ☆	Regulation covers three of the areas listed
	• A maximum blood alcohol content (BAC) at 0.05 g or less per 100 ml (or breath alcohol equivalent)<br>• Drink drive legislation sets a lower BAC for young drivers, compared with older drivers<br>• Random blood/breath alcohol testing is in place

Food

F1. Reducing salt consumption

Policies are in place to reduce population salt consumption
WHO Equivalent Indicator #7a

(red)	No salt reduction plans/activities are in place
(amber)	Salt reduction plans/activities are under development
(green)	Activities covers one of the areas listed
☆	Activities cover two of the areas listed
☆ ☆	Activities cover three of the areas listed
☆ ☆ ☆	Activities cover four of the areas listed
	• Salt reduction activities/objectives are articulated in NCD strategy or other relevant plan<br>• There is a stipulated population salt/sodium intake reduction goal<br>• Salt awareness programmes/education are in place<br>• Mandatory salt labelling regulations are in place<br>• Sodium targets are in place for food groups that are major contributors to sodium intake, based on international best practice

F2. Trans-fats

Policies are in place to limit trans-fats (i.e. partially hydrogenated vegetable oils) in the food supply
WHO Equivalent Indicator #7b

(red)	No trans-fats related policies/activities are in place
(amber)	There are no trans-fat prevention and control activities in place, but there is reference to trans-fats in relevant strategy or action plans (e.g. NCD plan, nutrition plan)
(green)	Activities cover one of the areas listed
☆	Activities cover two of the areas listed
☆ ☆	Activities cover three or four of the areas listed
☆ ☆ ☆	Activities cover five or six of the areas listed
	• Mandatory food labelling regulations that include total fats and trans-fats<br>• Ongoing monitoring of trans-fatty acids in processed foods and/or restaurants<br>• National dietary guidelines refer to reducing intake of trans-fatty acids<br>• Voluntary or mandatory controls on reuse of oils in catering establishments<br>• Awareness campaigns on trans-fat risks and avoidance are being conducted<br>• Mandatory food standards that prevent the sale of foods containing trans fats

F3. Unhealthy food marketing to children

Policies are in place to restrict marketing of unhealthy food to children
WHO Equivalent Indicator #7c

	There are no regulations in place to restrict promotion of unhealthy food to children
	Regulations are under development
	Some regulations are in place to limit 'unhealthy' (in line with WPRO nutrient profiling tool) food advertising/marketing to children, in one area listed
☆	Advertising/marketing is restricted in two or three areas listed
☆ ☆	Advertising/marketing is restricted in four or five areas listed
☆ ☆ ☆	Advertising/marketing is restricted in six or more areas listed
	• national television (times, channels)<br>• radio (times, channels)<br>• local magazines/newspapers (child-focused print, e.g. comics)<br>• billboards and outdoor advertising (near schools and early childhood education centres, at children-related events)<br>• through sponsorship for child-related events/sports<br>• advertising in settings where children gather: preschools, schools, school sports, school events, cultural events<br>• via packaging<br>• through free distribution of unhealthy products in areas where children gather<br>• at point of sale

F4. Food fiscal policies

Fiscal policies are in place to make healthy food choices easier and cheaper, and to discourage unhealthy food choices
WHO Equivalent Indicator: No equivalent

	Government has taken no specific measures to reduce the cost of healthy food or increase the cost of unhealthy choices
	Government is developing specific measures to reduce the cost of healthy food or increase cost of unhealthy choices
	Government has formulated specific measures to reduce the cost of healthy food and/or increase the cost of unhealthy choices in one area listed
☆	Government measures include two areas listed
☆ ☆	Government measures include three areas listed
☆ ☆ ☆	Government measures include four or five areas listed
	• Excise duties are levied on imported and/or locally sugar-sweetened beverages (SSB) of at least 20% of retail price; or fiscal import tax is imposed on raw materials for local producers to an equivalent level<br>• Provision is made to increase sugar-sweetened beverage taxation rates to account for inflation<br>• Fruit and vegetables are exempt from added taxes; and/or all unprocessed foods are zero rated VAT (or equivalent)<br>• Excise duties are levied on at least one imported/locally produced 'unhealthy food' (in line with WPRO nutrient profiling tool)<br>• The excise taxation system is stated by the government as an important public health tool to confront NCDs

F5. Healthy food policies in schools

Policies are in place relating to the provision and promotion of healthy food choices in schools
WHO Equivalent Indicator: No equivalent

	There are no government (Ministry of Health or Ministry of Education) policies or guidelines encouraging healthy food services in schools
	The Ministry of Health and/or Education is developing policies or guidelines to encourage healthy food services in schools
	There is a mandatory government policy or guideline for healthy food services in schools which covers one area listed
☆	There is a mandatory government policy or guideline which covers two areas listed
☆ ☆	There is a mandatory government policy or guideline which covers three areas listed
☆ ☆ ☆	There is a mandatory government policy or guideline which covers four areas listed
	• Healthy food/beverages are provided in school canteens<br>• Healthy food/beverages are sold in vending machines or school shop<br>• Healthy food/ beverages are used in fundraising<br>• Education and promotion of healthy food/beverage choices<br>• Healthy food/beverages at school events

F6. Food-based dietary guidelines

National food-based dietary guidelines are in place
WHO Equivalent Indicator: No equivalent

	There are no national food-based dietary guidelines for adults
	National food-based dietary guidelines for adults are under development, or a process is under way to adopt/adapt international or regional guidelines
	National food-based dietary guidelines for adults are in place, or international/regional guidelines have been adopted, that cover five of the areas listed
☆	National food-based dietary guidelines cover six of the areas listed
☆ ☆	National food-based dietary guidelines cover six of the areas listed<br><br>AND<br><br>Dietary food-based guidelines are included in the school curriculum
☆ ☆ ☆	National food-based dietary guidelines cover six of the areas listed<br><br>AND<br><br>Food-based dietary guidelines are included in the school curriculum<br><br>AND<br><br>There is evidence that food-based dietary guidelines are used to inform policy-making
	• Available in all principal languages<br>• Encourage consumption of a balanced diet<br>• Recommend the number of serves from each food group to be eaten each day<br>• Provide guidance about portion size<br>• Promote minimal consumption of fat, salt and sugar<br>• Promote physical activity and maintaining a healthy weight<br>• Promote healthy cooking practices<br>• Promote local food and traditional recipes<br>• Recommend exclusive breastfeeding for first six months and continued breastfeeding until at least two years of age

Physical activity

P1. Compulsory physical education in school curriculum

Physical education is a compulsory component of the school curriculum
WHO Equivalent Indicator #8

(red)	Physical education is not a specified element of the national school curriculum
(amber)	Physical education is identified as a key learning area of the national school curriculum but has no specific curriculum statement or syllabus<br>OR<br>Implementation of existing syllabus is not mandatory/enforced/monitored
(green)	Physical education is a key learning area of the national school curriculum, there is a curriculum statement or syllabus that covers at least levels K-10 (or equivalent), and implementation of the syllabus is mandatory and enforced in all schools
☆	As for (green), AND one of the areas listed
☆ ☆	As for (green), AND two of the areas listed
☆ ☆ ☆	As for (green), AND three of the areas listed
	The PE syllabus is mandatory for all pupils (no exclusions for students with disabilities, girls and those from minority groups)<br><br>The national PE curriculum statements / syllabus makes the relationship between physical exercise and health promotion clear and explicit to encourage a lifelong participation in physical activity<br><br>The Ministry of Education has budget allocated to support and develop PE teacher capacity and resources in schools (verbal report is sufficient evidence for this indicator)<br><br>The curriculum specifies a minimum of 30 minutes per day or three hours per week physical activity

Enforcement

E1. Enforcement of laws and regulations related to NCD risk factors

A system is in place to monitor and enforce laws and regulations related to NCD risk factors
WHO Equivalent Indicator: No equivalent

(red)	There is no organised system for enforcement of tobacco, alcohol, food (and betel nut if prevalent in the country) laws and regulations related to NCDs other than inspection of imports
(amber)	A government-level law and regulation enforcement system is planned for at least one NCD risk factor domain (tobacco, alcohol, unhealthy food and betel nut if prevalent in the country)
(green)	A government-level enforcement system is in place with retail and/or wholesale inspections documented within the past year for one NCD risk domain (tobacco, alcohol, NCD-related foods, betel nut). Note: Import inspections alone are not sufficient for green score.
☆	The enforcement system has had inspections documented within the past year and:<br>• includes two or more NCD risk domains (tobacco, alcohol, NCD-related foods, betel nut)<br>• there is a summary report available showing the compliance rate for each regulation surveyed
☆ ☆	The enforcement system has had inspections documented within past year and:<br>• includes three or more NCD risk domains (tobacco, alcohol, NCD-related foods, betel nut)<br>• there is a summary report available showing the compliance rate for each regulation surveyed
☆ ☆ ☆	The enforcement system has had inspections documented within past year and:<br>• includes three or more NCD risk domains (tobacco, alcohol, NCD-related foods, betel nut)<br>• there is a summary report available showing the compliance rate for each regulation surveyed<br>• at least some violators have been prosecuted and sanctioned (e.g. with fines)

	**Domain 1: Tobacco**<br><br>At least one of the following:<br><br>• Restrictions on tobacco advertising (see Indicator T4)<br>• Smoke-free environments (see Indicator T2)<br>• Tobacco sales, licensing and registration (see Indicator T5)<br>• Health warnings (see Indicator T3)<br>• Taxation (see Indicator T1)<br><br>**Domain 2: Alcohol**<br><br>At least one of the following:<br><br>• Restrictions on alcohol advertising (see Indicator A2)<br>• Taxation (see Indicator A3)<br>• Restrictions on alcohol sales (see Indicator A1)<br><br>**Domain 3: Food**<br><br>At least one of the following:<br><br>• Regulations on food labelling (trans fats, salt) and food standards (trans fats) (see F1 and F2)<br>• Controls on reuse of oils in catering establishments (F2)<br>• Restrictions on food marketing to children (F3)<br>• Food fiscal policies (see F4)<br>• Healthy food policies in schools (F5)<br>• Compulsory physical education in school curriculum (see P1)<br>• Marketing of breast milk substitutes (see H4)<br><br>**Domain 4: Betel nut**<br><br>• Restrictions on use of betel nut in workplaces or public spaces<br>• Betel nut sales to minors are banned<br>• Taxation

Health system response programmes

H1. National guidelines for care of main NCDs

National guidelines are in place for the diagnosis and treatment of the four main NCDs (cardiovascular disease, diabetes, cancer and chronic respiratory diseases) in public sector health facilities
WHO Equivalent Indicator #9

	No national guidelines exist for management of any of the four main NCDs in public sector health facilities
	National guidelines for some or all four of the main NCDs are under development, OR exist but are not implemented
	National guidelines for one of the four main NCDs are in place and are being implemented
☆	National guidelines are in place and implemented in public sector health facilities for two of the four main NCDs:<br><br>• Diabetes<br>• Cardiovascular disease (guidelines MUST include risk stratification)<br>• Cancer<br>• Chronic respiratory diseases
☆ ☆	National guidelines are in place and implemented in public sector health facilities for three of the four main NCDs
☆ ☆ ☆	National guidelines are in place and implemented in public sector health facilities for ALL four main NCDs

H2. Essential drugs

Essential NCD drugs are available and accessible in public sector primary care facilities
WHO Equivalent Indicator #10

	No essential drug list exists, or not all drugs listed below are on the essential drugs list
	All drugs listed below are on the essential drugs list
	All drugs listed are on the essential drugs list, and a system is in place to monitor availability
☆	As per , AND monitoring reports are available, AND stock-outs reported in more than 50% of primary care facilities in the last 12 months
☆ ☆	As per , AND monitoring reports are available, AND stock-outs reported in less than 50% of primary care facilities in the last 12 months
☆ ☆ ☆	As per , AND monitoring reports are available, and no stock-outs reported in primary health care facilities in the last 12 months
	• insulin<br>• aspirin (100 mg)<br>• metformin<br>• thiazide diuretics<br>• ACE inhibitors<br>• CC Blockers<br>• statins<br>• sulphonylureas

H3. Smoking cessation

Tobacco cessation support is available in all communities and is fully cost-covered
WHO Equivalent Indicator: No equivalent

	No cessation services available
	Cessation services are being developed
	Cessation services are available in at least one health care facility
☆	Cessation services (at a minimum, brief cessation intervention or 5A's) are available in at least one health care facility and cover one area listed
☆ ☆	Cessation services are available in at least one health care facility AND cover two areas listed
☆ ☆ ☆	Cessation services are available in at least one health care facility AND cover three or more areas listed
	• NRT available<br>• National Quitline<br>• Cessation services at all facilities<br>• Cessation services are fully cost-covered

H4. Marketing of breastmilk substitutes

National laws govern the implementation of the International Code of Marketing of Breastmilk Substitutes
WHO Equivalent Indicator #7d

	No government or self-regulated restrictions exist for marketing of breastmilk substitutes (BMS)
	Government regulations are under development according to the International Code of Marketing of BMS, or laws exist but are not implemented, or restrictions are self-regulated by the BMS industry
	Government regulations are in place and implemented according to the International Code of Marketing of BMS, and cover one area listed
☆	Regulations cover two areas listed
☆ ☆	Regulations cover three areas listed
☆ ☆ ☆	Regulations cover four areas listed
	• Regulations ban all forms of advertising or promotion of BMS to mothers and the general public. This includes point of sale advertising, free samples, discount coupons, and tie-in sales<br>• Regulations define products considered BMS to include infant formula, follow-on formula, bottles and teats, and complementary/weaning foods<br>• Regulations note that marketing of BMS is regulated to promote breastfeeding and ensure safe and adequate nutrition for infants and young children<br>• Regulations ensure that labels are designed to provide the necessary information about the appropriate use of the product, and not to discourage breastfeeding

H5. Baby-friendly hospitals

Government supports the Baby Friendly Hospital Initiative
WHO Equivalent Indicator: No equivalent

	No hospitals are Baby Friendly Hospital (BFH) certified, and none are working toward certification
	The BFH certification process has been adopted but no hospital has been externally BFH certified
	At least one public hospital has been BFH certified through external assessment
☆	More than 50% of public hospitals are BFH certified
☆ ☆	As for ☆, and all hospitals with BFH designation are monitored internally to keep track of current status (e.g. annually)
☆ ☆ ☆	As for ☆, and all hospitals with BFH designation are externally reassessed at intervals (e.g. five years)

H6. Maternity leave and breastfeeding

Legislation is in place providing maternity leave and breastfeeding breaks/facilities
WHO Equivalent Indicator: No equivalent

	There is no legislation for maternity leave
	Legislation for maternity leave is under development or does not meet the standard required for green rating
	Legislation is in place providing at least 12 weeks paid maternity leave, with the mother paid no less than two-thirds of her previous earnings
☆	As for , AND legislation is in place covering one of the following areas:<br>• Provision of breast-feeding facilities in workplaces and/or public areas<br>• Provision of breast-feeding breaks for working mothers<br>• Provision of at least 14 weeks paid maternity leave, with the mother paid no less than two-thirds of her previous earnings
☆ ☆	As for , AND legislation is in place covering two of the areas listed
☆ ☆ ☆	As for , AND legislation is in place covering three of the areas listed

Monitoring

M1. Population risk factor prevalence surveys - adults

A population NCD risk factor prevalence survey for ADULTS has been conducted in the last 5 years which includes physical and biochemical measurements
WHO Equivalent Indicator #3

	Risk factor prevalence data more than ten years old
	Risk factor prevalence data five to ten years old and survey scheduled in the next 18 months
	Risk factor prevalence data collected within the last five years
☆	The survey data collected include at least three of the risk factors listed
☆ ☆	The survey data collected within the last five years includes six or more of the risk factors listed
☆ ☆ ☆	The survey data collected within the last five years includes all of the factors listed below AND there is intention for regular future surveys (every one or two years, or three to five years)
	• Harmful use of alcohol<br>• Physical activity<br>• Tobacco use<br>• Raised blood glucose/diabetes (objective measurement)<br>• Raised blood pressure/ hypertension (objective measurement)<br>• Obesity and overweight (physical measurement)<br>• Salt/sodium intake (objective measurement, e.g. spot urine sample)

M2. Population risk factor prevalence surveys - youth

A population NCD risk factor prevalence surveys for ADOLESCENTS (13–17 years) has been conducted in the last two years which includes physical measurements for NCDs
WHO Equivalent Indicator: No equivalent

	Risk factor prevalence data more than five years old
	Risk factor prevalence data more than five years old and survey scheduled in the next 12 months
	Risk factor prevalence data reported within the past three to five years
☆	Risk factor prevalence data reported within the past three to five years and:<br>• includes physical measurement of overweight and obesity<br>• repeat survey scheduled in the next 12 months
☆ ☆	Risk factor prevalence data reported within the past two years and:<br>• includes physical measurement of overweight and obesity
☆ ☆ ☆	Risk factor prevalence data reported within the past two years and:<br>• includes physical measurement of overweight and obesity<br>• includes at least three of the following risk factors: alcohol use, physical activity, tobacco use, betel nut use, dietary information (at least one indicator)

M3. Child growth monitoring

Childhood growth data (age 3-12 years) is routinely monitored and reported

	No growth data collected for children less than 13 years of age
	Some childhood growth data are collected but not reported
	Childhood growth data are collected and reported
☆	As for , and two of the items listed
☆ ☆	As for , and three of the items listed
☆ ☆ ☆	As for , and four of the items listed
	• Data collected for more than one age/grade<br>• Dataset is available to within-country stakeholders (e.g. other ministries) for analysis<br>• Data reported at least every two years<br>• Training/standardisation of height and weight measurement<br>• Extra risk factor data are collected (e.g. nutrition, physical activity)

M4. Routine cause-specific mortality

There is a functioning system for generating reliable cause-specific mortality data on a routine basis
WHO Equivalent Indicator #2

	A basic vital registration system is not in place (basic system must have all of the following elements: captures deaths; certifiers complete the International Form or Medical Certificate of the Cause of Death; and International Certification of Diseases (ICD) is used to code deaths)
	Vital registration is in development
	A vital registration system exists, and cause of death data are compiled and publicly reported.
☆	As for , and one of the items listed
☆ ☆	As for , and two of the items listed
☆ ☆ ☆	As for , and three of the items listed
	• At least five years of cause-of-death data have been reported<br>• The most recent year of data reported is no more than five years old<br>• Reliable reporting from outlying districts (e.g. outer islands)
